# Cyclopeptide COR-1 to treat beta1-adrenergic receptor antibody-induced heart failure

**DOI:** 10.1371/journal.pone.0201160

**Published:** 2018-08-20

**Authors:** Valérie Boivin-Jahns, Kerstin Uhland, Hans-Peter Holthoff, Niklas Beyersdorf, Vladimir Kocoski, Thomas Kerkau, Götz Münch, Martin J. Lohse, Martin Ungerer, Roland Jahns

**Affiliations:** 1 Department of Pharmacology and Toxicology, University of Würzburg, Comprehensive Heart Failure Centre (CHFC), University Hospital Würzburg, Würzburg, Germany; 2 Procorde, Martinsried, Germany; 3 Institute for Virology and Immunobiology, University of Würzburg, Würzburg, Germany; 4 Interdisciplinary Bank of Biomaterials and Data Würzburg, Comprehensive Heart Failure Centre (CHFC), Würzburg, Germany; Central University of Tamil Nadu, INDIA

## Abstract

**Rationale:**

Despite advances in pharmacotherapy, heart failure still incurs significant morbidity and mortality. Stimulating antibodies directed against the secondextracellular loop of the human ß_1_-adrenergic receptor (anti-ß_1_EC2) cause myocyte damage and heart failure in rats. This receptor domain is 100% homologous between rats and humans.

**Objective:**

ß_1_EC2-mimicking cyclopeptides (25-meric) markedly improved the development and/or course of anti-ß_1_EC2-mediated cardiomyopathy. Further developments should be investigated.

**Methods and results:**

The shortened 18-meric cyclic peptide COR-1, in which one of the two disulphide bonds was removed to enable reproducible GMP production, can also be used to treat cardiomyopathic rats. Echocardiography, catheterization and histopathology of the rat hearts revealed that monthly intravenous administrations of COR-1 almost fully reversed the cardiomyopathic phenotype within 6 months at doses of 1 to 4 mg/kg body weight. Administration of COR-1 resulted in markedly reduced anti-ß_1_EC2-expressing memory B lymphocytes in the spleen despite continued antigenic boosts, but did not significantly decrease overall peripheral anti-ß_1_EC2 titers. COR-1 did not induce any anti-ß_1_EC2 or other immune response in naïve rats (corresponding to findings in healthy human volunteers). It did not cause any toxic side effects in GLP studies in dogs, rats or mice, and the “no observed adverse effect level” (NOAEL) exceeded the therapeutic doses by 100-fold.

**Conclusion:**

The second generation immunomodulating epitope-mimicking cyclopeptide COR-1 (also termed JNJ-5442840) offers promise to treat immune-mediated cardiac diseases.

## Introduction

Heart failure (HF) is a life-threatening syndrome characterized by shortness of breath, fluid retention, and reduced cardiac function. Despite recent advances in pharmacotherapy, about 50% of patients die within four years[1]. One key player in the regulation of cardiac function is the beta1-adrenergic receptor (ß_1_-AR) situated in the membrane of cardiomyocytes. Upon physical or psychical stress ß_1_-AR transmit some of the effects of catecholamines to the heart[2–4]. Whereas short-term adrenergic stimulation serves to temporarily improve cardiac performance on demand, chronic activation of the sympathetic nervous system has the opposite effect, and over time leads to progressive deterioration of cardiac structure and function[5].

Several studies have shown that many heart failure patients exhibit catecholamine-like acting autoantibodies directed against the cardiac ß_1_-AR (anti-ß_1_–abs)[6–9]. Such receptor-stimulating anti-ß_1_–abs are particularly found in patients with idiopathic dilated cardiomyopathy (DCM), a non-ischemic heart muscle disease of unknown etiology characterized by dilatation and impaired contraction of the left ventricle[10]. Clinically, the presence of stimulating anti-ß_1_–abs has been associated with a more severely reduced cardiac function[11], a higher incidence of life-threatening ventricular arrhythmias and sudden cardiac death[12], and an increased cardiovascular mortality risk[13]. However, efficient and specific therapeutic strategies to combat these harmful receptor-antibodies are still lacking. Most functional anti-ß_1_–abs were shown to target the second extracellular loop of the ß_1_-AR protein (ß_1_EC2), representing the largest of in total three EC-loops and, thus, a readily accessible target on the cell surface[7,14]. Moreover, ß_1_EC2 contains T- and B-cell epitopes[15] turning it into a potent self-antigen. The receptor’s crystal structure suggests that ß_1_EC2 is essential for the stabilization and locking of the receptor’s catecholamine-binding pocket[14,16]. Thus, it seems conceivable that conformational anti-ß_1_EC2–abs may allosterically increase ß_1_-receptor activity[7,17]. Monthly immunization of Lewis rats with fusion proteins containing ß_1_EC2 gives rise to stimulating anti-ß_1_EC2–abs. Within 9 months anti-ß_1_EC2–positive rats develop progressive left ventricular dilatation, wall thinning, and downregulation of cardiac ß_1_-AR,a feature typical for human DCM [6,18,19]. We found that ß_1_EC2–mimicking cyclopeptides given either (a) shortly after the induction of stimulating anti-ß_1_EC2–abs or (b) in overt heart failure strongly improved the development and/or course of heart failure[20]. They were more efficient than the clinically used ß_1_-AR receptor blocker bisoprolol[20].

In this follow-up study, we investigated whether the novel cyclic peptide COR-1 (also termed JNJ-5442840) also improves important functional and immunological parameters which characterise autoimmune heart failure. We also tested COR-1 effects on naïve animals, and potential side effects in comprehensive toxicological and pharmacokinetic studies.

## Materials and methods

### Generation and characterization of ß_1_-EC2-homologous cyclopeptides

Cyclic peptides (CP) were synthesized by Polypeptide, Strasbourg, France according to described protocols of fluorenylmethoxycarbonyl (FMOC) resin-based amino acid chain elongation, and subsequent head-to-tail cyclisation. Fmoc-Asp(OBut)-(Dmb)Gly-OH was attached to a 2-chlorotrityl chloride resin (MERCK/NOVA BIOCHEM) yielding a resin of 0,30 mmol/g. Peptide synthesis was done by a standard cycle of deblocking with 30% piperidine/ N,N-dimethylformamide (DMF) (5+12 min) and coupling with 3 eq. Fmoc-amino acid/TBTU/6 eq. N-methylmorpholine (NMM) in DMF (double coupling, 2 x 30 min). After cleavage from the resin by 20% hexafluoroisopropanol (HFIP)/DCM (2 x 20 min), the isolated crude peptides were cyclized by 3 eq 7-Azabenzotriazol-1-yloxy)tripyrrolidinophosphonium hexafluorophosphate (PyAOP)/ 5 eq. diisopropylethylamine (DIEA) in DMF overnight, the solvent was evaporated and the crude peptides were deblocked by trifluoroacetic acid (TFA)/water/ thioanisol (TIS) (95:5: 3) in 2h. Then, the peptides were purified up to 95% by means of HPLC and analyzed by MALDI-TOF mass spectrometry. Intramolecular disulphide bridges between cysteins form spontaneously and reproducibly at these conditions.

The generated cyclopeptide ß_1_EC2-CP was biochemically analyzed by high pressure liquid chromatography (HPLC), and by mass spectroscopy (MALDI). HPLC was carried out in a Waters Separation Modul 2690 together with a Waters Dual Lambda absorbance detector; absorbance was read at 220 nm. After peptide-synthesis and cyclization, the samples were dissolved in H_2_O/5% acetonitril (ACN) and loaded on a Nuclosil 100-5/ C18 column (Macherey-Nagel Inc., Germany; column length 250 mm, lumen 4 mm) applying a flow of 1 ml/min and a separation-gradient from 5 to 60% ACN in the presence of 0.2% TFA. The remaining faint amount of non-cyclized ß_1_EC2-peptide yielded a small peak, typically detected between 14 and 16 min, whereas the fractions containing ß_1_EC2-CP appeared in a range from 18 to 22 min. Aliquots of these fractions containing 20–80μg/ml of ß_1_EC2-CP were further analyzed by mass spectroscopy, and dissolved in phosphate-buffered saline (PBS).

### Surface plasmon resonance (SPR)

SPR measurements were carried out at Biaffin GmbH, Kassel, Germany, using about 1350 RU immobilized monoclonal antibody 23-6-7 and concentrations of COR-1 ranging from 0.1 nmol/L to 1 μmol/L in 20mM HEPES pH 7.4, 150mM NaCl, 0.005% Tween p20, at a flow of 30μl/min at 25°C.

### ELISA using peptide coating and detection of the ß_1_-AR EC2-specific monoclonal antibody 23-6-7

Concentrations of COR-1 in rat plasma were determined by a competitive enzyme-linked immunosorbent assay (ELISA) in which the 16-meric ß_1_EC2 peptide competed with soluble COR-1 for binding of the ß_1_EC2-specific antibody 23-6-7.

First, streptavidin-coated ELISA plates were incubated with C-terminally biotinylated ß_1_EC2 peptides. Saline, plasma, serum, or whole blood, respectively, spiked with various concentrations of COR-1 and with defined concentrations of the anti-ß_1_EC2 antibody, respectively, were then added onto the plates. Then, the plates were incubated with a secondary antibody with specificity to the IgG of the primary antibody conjugated with horseradish peroxidase (POD) to detect bound antibodies. A chromogenic peroxidase substrate was added and adsorption was measured using an ELISA reader.

### ELISA using antibody coating and detection of DEARR-Bio-16 mer

The inhibitory concentration (IC_50_) of COR-1 to mAb 23-6-7 was determined by a competitive enzyme-linked immunosorbent assay (competitive ELISA) in which the biotinylated 16-meric ß_1_EC2 peptide DEARR-Bio competed with soluble COR-1 for binding to mAb 23-6-7. IC_50_ values were determined at three different fixed concentrations of the biotinylated peptide, at 10, 3.3, and 1 nM, respectively.

In contrast to the competitive ELISA used to determine COR-1 concentrations, where the biotinylated 16-meric peptide was coated onto the ELISA plates, the mAb 23-6-7 was coated onto the plate and the biotinylated 16-meric peptide and COR-1 were used in solution.

First, protein G-coated ELISA plates were incubated with mAb 23-6-7. The plates were blocked and then incubated with fixed concentrations of the biotinylated 16-meric peptide as well as with various concentrations of COR-1. The bound 16-meric peptide was then labelled by Streptavidin- POD conjugate and detected by a chromogenic peroxidase substrate. The adsorption was measured using an ELISA reader.

### Cytokine measurements

Rat interleukin-6 (IL-6) and tumour necrosis factor (TFN)-alpha concentrations were determined by commercially available ELISA kits (Thermo Scientific ER2IL6 kitand ER3TNFA, both from Thermo Scientific).

### Animal experiments and study protocol

**Fig 1:**
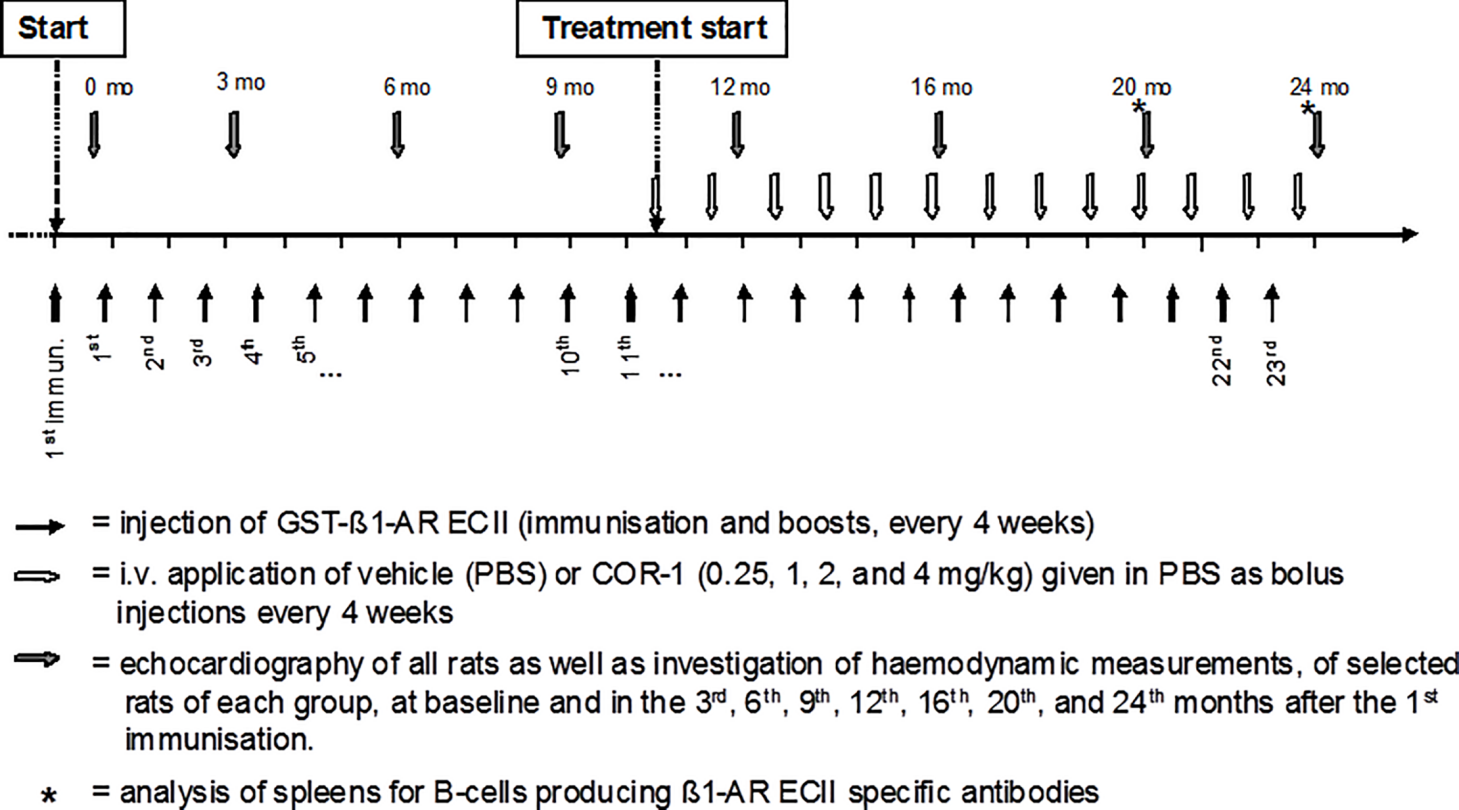
Study protocol (rat experiments).

The study protocol of the main efficacy study, the time lines of immunisations and therapies are outlined in Fig 1. This protocol, as well as further pharmacokinetic and–dynamic studies and guideline-conform animal housing conditions were approved by the local authorities. Studies performed at the University of Würzburg were approved by the Experimental Animal Use and Care Committee, Government of Lower Franconia (vote No. 621–2531.01-35/04), and studies performed at Martinsried were approved by the local animal welfare authority and Ethics committee at the Government of Upper Bavaria in Munich, Germany (vote no. 55.2-1-54-2531-25-12).

Also, safety and toxicity studies at external GLP service providers werecarried out after approval by the respective local authorities. All animal studies were in accordance to the World Medical Association (Declaration of Helsinki), and the European Commission guidelines (Directive 2010/63/EU).

### Generation and characterization of anti-ß_1_EC2-antibodies

Fusion proteins of glutathion-S-transferase (GST) and the second extracellular loop of the human ß_1_-AR (ß_1_EC2; AA195-225[3]) served to s.c. immunizeLewis/CrlBR rats every month over 24 months as described previously[19, 20, 21]. Rat serum was assayed for reactivity by sandwich ELISA with 25-meric linear peptides corresponding to the human ß_1_EC2-sequence used for coating and a combination of biotinylated anti-rat IgG antibody and Streptavidin-POD for detection of ß_1_EC2-specific bound rat IgG.

### Echocardiography

Transthoracic echo-Doppler examinations were performed as previously described[19, 20]by the same experienced echocardiographer blinded to the treatment groups. In brief, the rats were lightly anaestetized (30 mg/kg ketamine-HCl and 5 mg/kg xylazine i.p.), shaved (chest only), and placed on a special table. Echocardiograms were obtained by a commercially available echocardiographic system (Vevo770, Visual Sonics Inc., Dallas, USA) equipped with a 17.5 MHz transducer. M-mode tracings were recorded at baseline (before immunization), and subsequently every 3 months in the parasternal long and short axis views according to the guidelines of the American Society for Echocardiography. Pulsed-wave Doppler spectra were recorded from the apical five-chamber view and the velocity-time integral (VTI) of the aortic outflow served to calculate cardiac output (CO [ml/min] = Aortic VTI x (π [LV-outflow tract diameter/2]^2^) x heart rate). LV-mass was assessed by using a modified cube formula equation. Reproducibility of M-mode and Doppler measurements was assessed as previously described[19]; intra- and interobserver variabilities were <2 or <5%. In addition, all final echocardiograms were validated anatomically.

### Hemodynamic measurements

Fourty-eight to 72 hours after the final echo-Doppler examinations the rats underwent left heart catheterization. The rats were lightly anaestetized as described above and a 2.5 F high-fidelitycatheter (Millar Instruments, Houston, Texas) was inserted via the right carotid artery into the left ventricle. LV-pressure tracings were recorded digitally over 15 min and analyzed off-line (PowerLab, A.D. Instruments, Castle Hill, Australia). After registration of the hemodynamic parameters 2 ml of blood were drawn from each animal to determine (final) anti-ß_1_EC2–titers and serum routine laboratory parameters. After additional deep anesthesia (70 mg/kg sodium pentobarbital i.p.) animals were euthanized, and the hearts were quickly removed, rinsed with ice-cold relaxing buffer (5% dextrose, 25 mM KCl in PBS), and weighed (wet weight). The apical half and a 2 mm slice from the upper half (always taking the aortic valve as a reference) was cut, frozen in isopentane (-56°C), and stored at -80°C for further analyses.

### ELISpot assays

ELISpot assays (Enzyme Linked Immuno Spot Assay) were carried out with B-cells prepared from either the spleen or the bone marrow of immunized anti-ßß_1_EC2-positive untreated animals compared with COR-1-treated animals. For the assays, ELISpot plates were coated overnight with either 1.8 μg/ml anti-rat IgG (H+L) or the specific antigen (GST/ß_1_EC2-FP) in 0.05 mol/l Tris buffer, pH 9.4. Then the plates were washed 3 times and blocked with BSA for 1 hour at 37°C. Subsequently, the plates were incubated overnight at 37°C with B-cells from either spleen or bone marrow (cultured in RPMI 1640/X-VIVO-15 medium supplemented with 10% fetal calf serum (FCS)) with 1x10^6^ to 1x10^3^ cells per well. After 16 hours the B cells were discarded and the plates with the B cell-secreted IgG bound were washed several times (PBS/0.5% Tween) before the addition of alkaline phosphatase conjugated secondary anti-rat IgG (0.3 μg/ml) to detect bound rat IgG. Then the plates were incubated for another 3 hours at 37°C, washed several times with PBS/0.5% Tween, and developed using LMP/BICP 5:1 (1 ml per well; LMP, low melting agarose; BICP, 5-bromo-4-chloro-3-indolyl phosphate *p*-toluidine salt, a chromogenic substrate for alkaline phosphatase) allowing for a quantification of the blue spots, with each spot representing either an IgG or an antigen-specific IgG secreting spleen or bone-marrow cell, respectively.

### Cardiac mRNA expression levels

Total RNA was isolated from myocardium using the SV total RNA isolation system (Promega, Madison, WI, USA), according to the manufacturer’s instructions. RT reactions were performed using a Taq Man Gold RT-PCR Kit (Applied Biosystems, Foster City, CA, USA). Random hexamers were used as primers for the RT reaction. The cycling parameters were as follows: 10 min at 25°C, 30 min at 48°C and 5 min at 95°C. Real-time PCR analyses for ß_1_-AR transcripts were performed with Taq Man assay-on-demand on the ABI 7700 Sequence Detection System, according to the manufacturer’s recommendations. The sequences of primer/probe of ß_1_ARwere as follows (5’−3’): ß_1_-ARsense: TGCAGACGCTCACCAACCT; ß_1_-ARanti-sense: CAGCAGTCCCATGACCAGATC; ß_1_-ARFAM-MGB probe: TTCATCATGTCCCTGGCC. The reactions were analyzed in triplicate and the relative expression levels were calculated according to the standard curve method. The expression data were normalized to an endogenous control, glyceraldehyde-3-phosphate-dehydrogenase (GAPDH). The expression was determined as the ratio of ß_1_-AR RNA /GAPDH RNA.

### Investigation of immunologically naïve animals

The effects of COR-1 were also assessed in naive, non-immunized rats to exclude antigenecity or general immune responses to COR-1. Male Lewis HanHsd rats were treated with 0.25 to 5 mg/kg COR-1 by intravenous bolus injection once every 4 weeks, for a total of six months. The formulation of COR-1 was identical to the previous studies, in PBS without further additives. Six rats were included in each group and assessed independently. Anti-COR-1 titers were measured in plasma samples taken either prior to and 24h and two weeks after COR-1 or vehicle injection by a sandwich ELISA with coated 25-meric ß_1_EC2 peptide (see above) and anti-rat IgG antibody-POD conjugate to detect bound ß_1_EC2-specific antibodies.

### GLP safety studies of toxicology and safety pharmacology

With exception of the in vivo part of safety study 1, all studies described in the following were conducted in compliance with GLP principles at GLP-conforming contract labs in several species:

Safety study 1: The long-term effect of higher doses of COR-1 was investigated in rats with HF due to prior immunization with the GST-ß_1_EC2 fusion protein. With ongoing monthly immunizations, 10 male and 10 female rats were given 30 mg/kg COR-1 by intravenous injection every four weeks for six months; another 10 rats received 1 mg/kg every four weeks for three months, and were observed without further therapy for another three months. Additionally, 10 rats were treated with vehicle for three months, and were observed without further therapy for another 3 months.

Safety study 2: Six male and six female Hsd Wistar rats were given two injections at dose levels of 25, 50 and 100 mg/kg COR-1 or vehicle. The first dose was applied on day 0, and the second dose on day 14, followed by a treatment-free period of another 14 days. Clinical examinations were carried out once a day. All animals were sacrificed and necropsied on day 28 and examined for macroscopic pathological changes.

Safety study 3: Three male and four female healthy beagle dogs were given doses of 10 or 20 mg/kg COR-1 seven-fold or 100 mg/kg once, then 6-fold 30 mg/kg COR-1, or vehicle. The first administrations were given as an infusion over 1 hour, the following dosing were given every second day as i.v. bolus injections. Additionally, a set of four animals was treated with 20 mg/kg, and observed for a further 14-day treatment free period after the end of the dosing period (“recovery group”). Full ECG documentation and blood pressure was recorded during the first infusion. Clinical examinations were carried out in all animals three times a day. Animals were sacrificed and necropsied on day 14 (main study animals) or day 28 (recovery animals). Analysis of haematologic routine parameters as well as standard clinical chemistry was carried out.

Safety study 4: Groups of four male and four female healthy beagle dogs were given 7.5, 15 or 30 mg/kg COR-1, or vehicle, once monthly over six months. The first dose was applied on day 0 and the following doses each subsequent month. For these four groups the overall study lasted for 7 months. Additionally, a set of four animals was treated with 30 mg/kg, and was observed for a further 2 months treatment free period after the end of the dosing period (“recovery group”). Clinical findings were investigated daily. In addition, ophthalmoscopy, measurements of blood pressure, pulse rate and ECG as well as routine laboratory (haematology and clinical chemistry) was carried out. 42 organs from each animal were also carefully analysed by histology.

### Statistical analysis

Data are shown as mean±SEM. Significance between the treatment groups was analyzed by ANOVA, followed by Scheffé´s F test. Comparisons between the cardiovascular effects upon injection of the different peptides, and comparisons between echocardiographic parameters (long-term follow-up) were done by repeated measures ANOVA accompanied by a Bonferroni post-hoc test. Agreement between the echocardiographic measurements (intra- and interobserver variability) was assessed. Hemodynamic and morphometric parameters of antibody-positive and corresponding control rats were compared by (unpaired) Student‘s t-test. Values of *P* < 0.05 were considered statistically significant.

## Results

### Cyclic peptide COR-1

**Fig 2:**
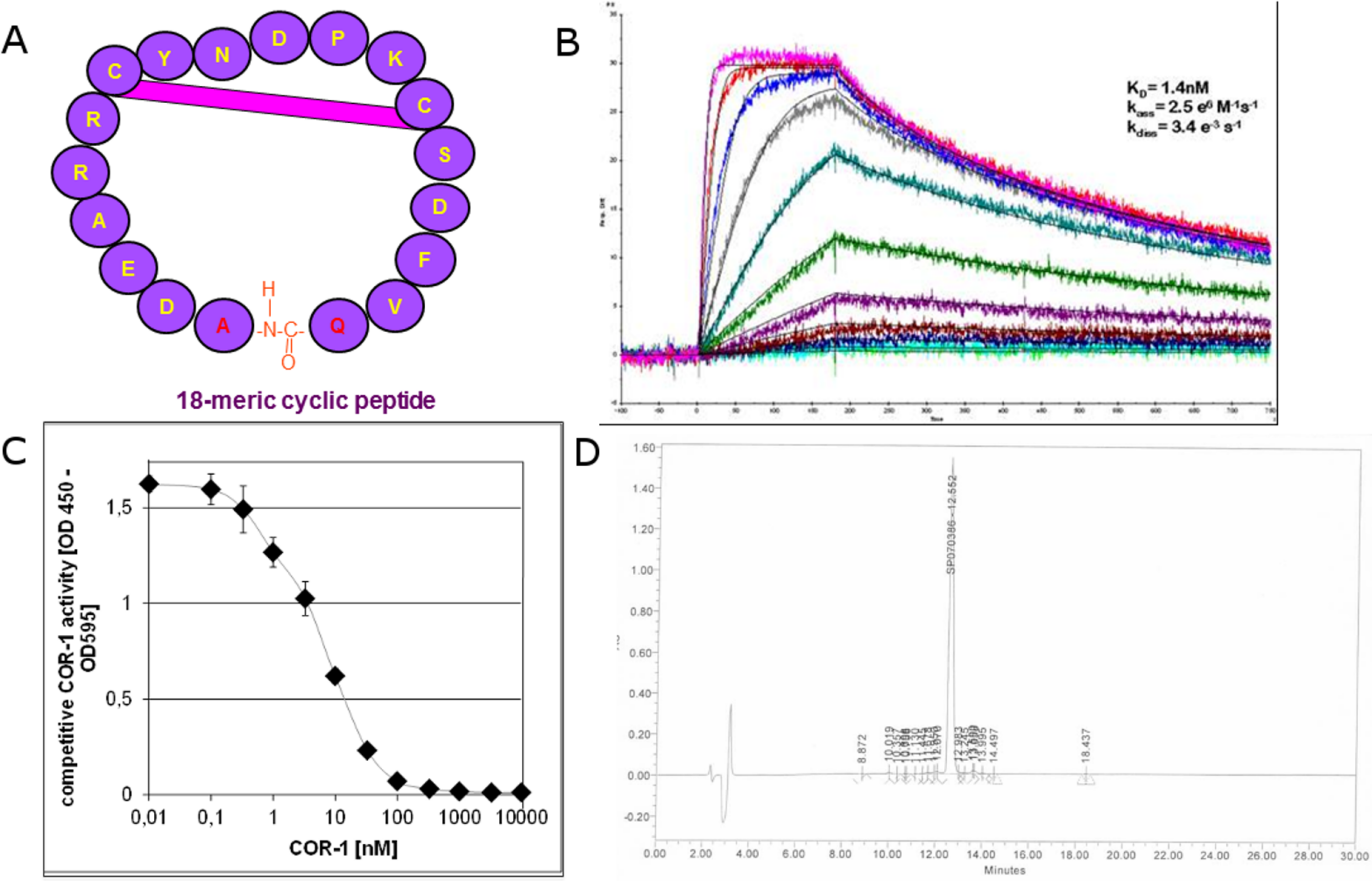
**A. Cartoon demonstrating the structure of COR-1**. **B: Surface plasmon resonance.** Representative tracing of the quantitative analysis of the interaction of COR-1 with the anti-ß_1_EC2–antibody 23-6-7 by surface plasmon resonance. Concentrations of 0.125 nmol/L to 128 nmol/L COR-1 were investigated at 30μl/min and 25°C. **C: ELISA:** Inhibition of binding of biotinylated 16-meric ß_1_EC2 peptides to the coated anti-ß_1_EC2 antibody 23-6-7 in the presence of increasing concentrations of COR-1 **D: HPLC profile:** HPLC shows one sharp peak of COR-1 and only few side products. COR-1 purity exceeds 95%.

Compared to previously used 25-meric cyclic peptides derived from the 2^nd^ extracellular loop of the ß_1_-AR[20],COR-1 was shortened to 18 amino-acids (AA) as shown in Fig 2A. One out of the three cysteine residues was replaced by serine, so that one instead of two intramolecular disulphide bonds are formed in this cyclic peptide. COR-1 was first synthesized as a linear peptide, and was then cyclized covalently on the backbone by condensation of the C-terminal carboxyl group with the amino group of the N-terminal amino acid. Subsequently, a disulphide bond between cysteine residues 7 and 13 formed spontaneously.

The predicted molar mass of COR-1 is 2097.3 Da. The experimental molecular weight of COR-1 determined by mass spectrometry was 2097.8 Da and therefore almost identical to the predicted weight. Liquid chromatography (HPLC) showed good purity exceeding 95% (see Fig 2D).

**Fig 3:**
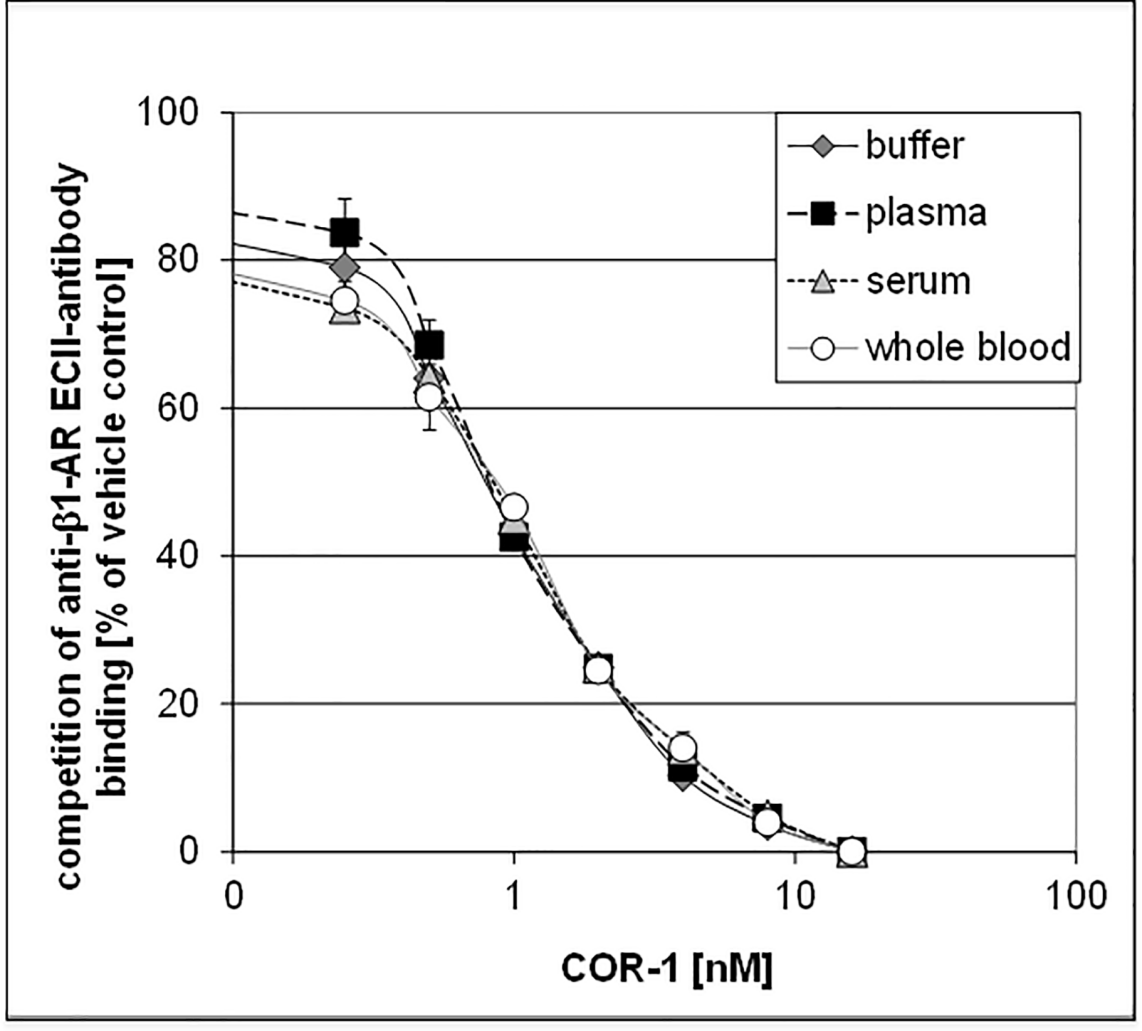
ELISA: Inhibition of anti-ß_1_EC2–antibody binding to coated 25-meric ß_1_EC2 peptides in the presence of increasing concentrations of COR-1, on a background of either 25% saline or rat serum, rat plasma or rat whole blood.

Affinity of COR-1 to the prototypical monoclonal anti-ß_1_EC2 antibody 23-6-7^22^ was assessed by surface plasmon resonance (Biacore) and by ELISA (Fig 2B and 2C). Both assessments resulted in similar, nanomolar affinity values. COR-1 affinity was not relevantly altered in the presence of various human blood fractions (Fig 3).

### The cyclopeptide COR-1 reverses anti-β_1_EC2-induced heart failure

Rats were immunized with ß_1_EC2/GST–fusion proteins every month. After 9 months, anti-ß_1_EC2–positive rats developed left ventricular (LV) dilatation and dysfunction, which continuously progressed with ongoing immunisations. Ten months after the first immunisation-boost and successful induction of anti-ß_1_EC2–abs the animals received monthly injections of 0.25–4 mg/kg of COR-1, or no specific intervention (positive control, vehicle). Fig 1 depicts the study protocol. Cardiac function was followed every 4 months by echocardiography, and invasively assessed at the end of the study as described[19,20].

**Fig 4:**
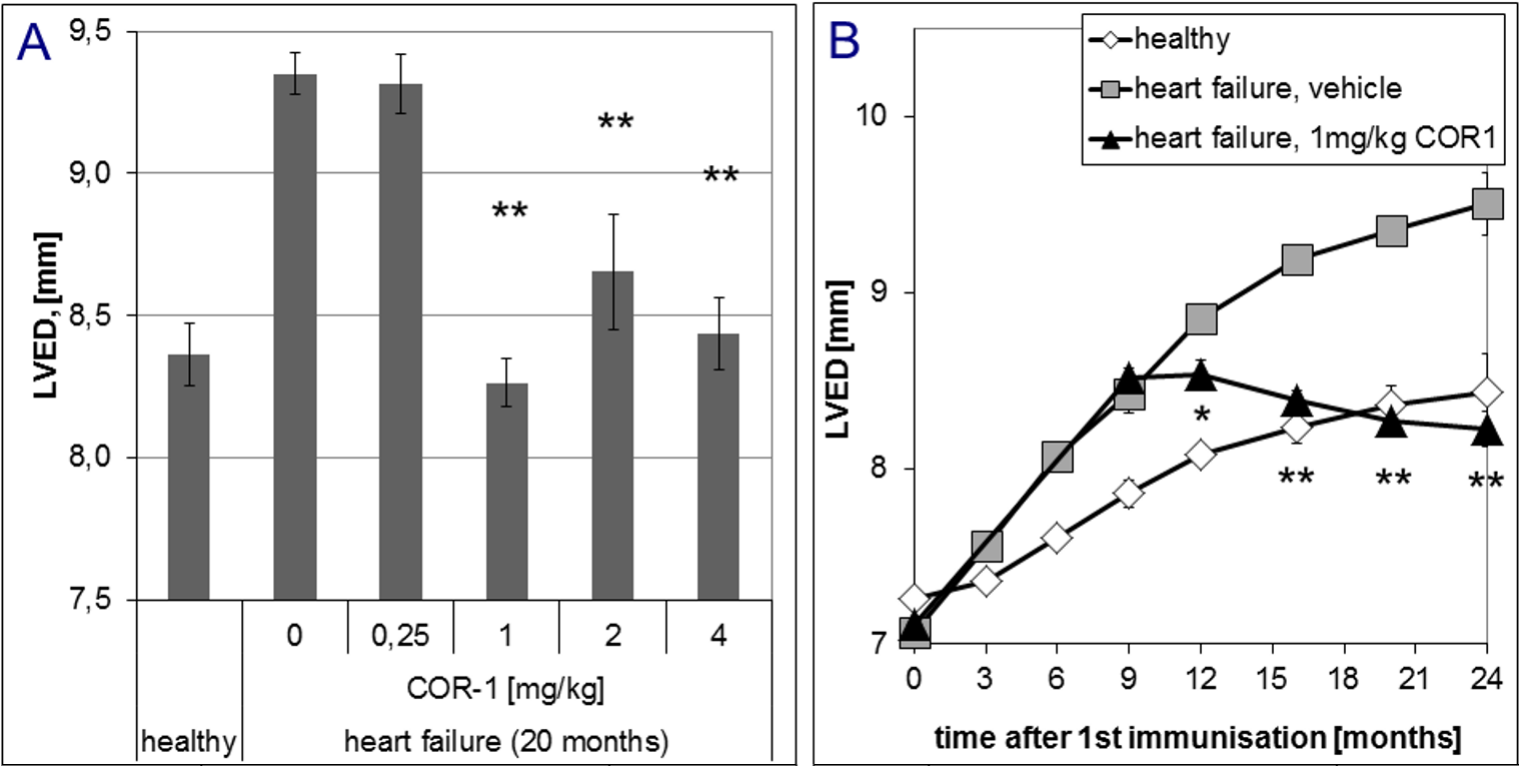
Effect of COR-1 on the left ventricular end-diastolic diameters (LVED) of rats with HF induced by immunisation with GST-ß_1_EC2 fusion proteins. **A**: Effects of COR-1 on LVED (with SEM)of immunized rats after ten treatments (20 months after the first immunization), including the ß_1_EC2HF vehicle control group (COR-1 0 mg/kg; n = 8), the ß_1_EC2HF groups treated with 0.25 mg/kg (n = 4), 1 mg/kg (n = 20), 2 mg/kg (n = 5) or 4 mg/kg BW COR-1 (n = 9) vs. healthy control animals (n = 9).The resulting ANOVA analyses showed overall significance, and post-hoc specific inter-group p values were 0.000 for HF + vehicle vs. healthy, 0.002 for HF + 0.25 mg/kg COR-1 vs. healthy, and 0.000 for HF + 1 mg/kg COR-1 vs. HF + vehicle, 0.032 for HF + 2 mg/kg COR-1 vs. HF + vehicle, 0.000 for HF + 4 mg/kg COR-1 vs. HF + vehicle. All other post-hoc analyses yielded non significant results. **B:** Time course of LVED obtained by echocardiography from healthy rats (white diamonds) and ß_1_EC2HF rats treated with vehicle (grey squares) or 1 mg/kg BW COR-1 (black triangles)every four weeks starting 10.5 months after the first immunization. At least four animals were analyzed independently per group and time point.* indicates statistical significance (p<0.05), and ** indicate strong statistical significance (p<0.005),when compared to the vehicle group with heart failure. Analysis of variance followed by Scheffe´s post-hoc test showed no difference at baseline, but significant (p < 0.005) worsening of LVED in HF rats compared to healthy control rats after 3 and 6 months (and all time points thereafter). HF rats treated with 1, 2 or 4 mg/kg BW COR-1 differed significantly (p<0.005) from the HF vehicle group, and did not differ from healthy control rats at 12, 16, 20 and 24 months (groups with 2 mg/kg and 4 mg/kg BW not shown to improve clarity of the image).

**Fig 5:**
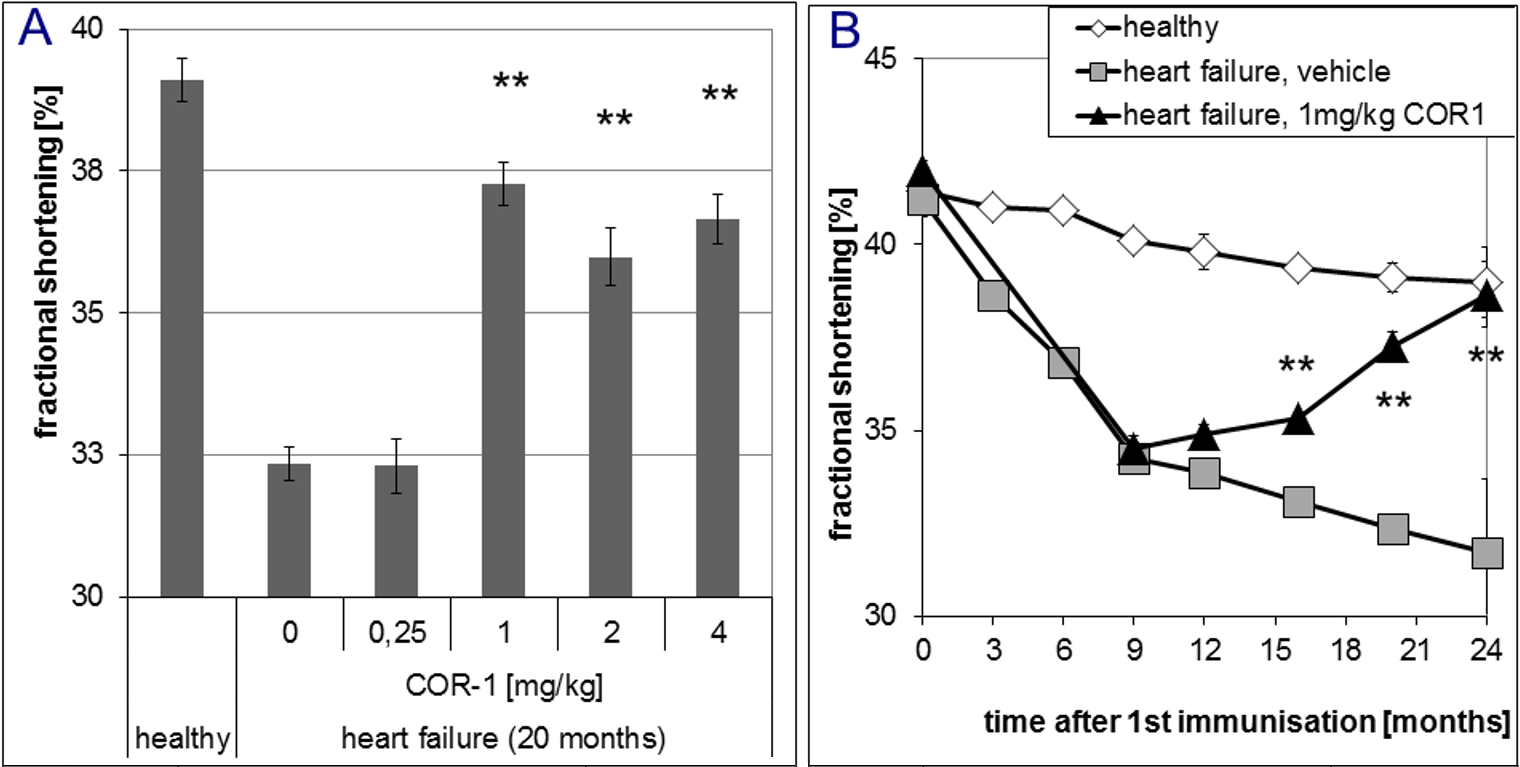
Effects of COR-1 on the fractional shortening (FS) of rats with HF induced by immunisation with GST-ß_1_EC2 fusion proteins. **A:** Effects of COR-1 on the FS after ten treatments (in the 20^th^ month after the first immunization), as determined by echocardiography. Mean FS with SEMs are shown for the vehicle control group (n = 8) compared to the ß_1_EC2HF groups treated with 0.25 (n = 4), 1 (n = 20), 2 (n = 5) or 4 mg/kg BW COR-1 (n = 9), respectively. For comparison, mean FS are also shown for healthy control rats(n = 9). ** indicates strong statistical significance (p<0.005) compared to the HF vehicle control group. **B:** Time course of the fractional shortening (FS), as determined at intervals of three months. Mean FS with SEMs of healthy rats (white diamonds) and of HF rats treated with vehicle (grey squares)every four weeks starting 10.5 months after the first immunisation, or with 1 mg/kg BW COR-1 (black triangles). At least four animals were analyzed independently per group and time point. *indicate statistical significance (p<0.05) and **indicate strong statistical significance (p<0.005), respectively, when compared to the HF vehicle group.

**Fig 6:**
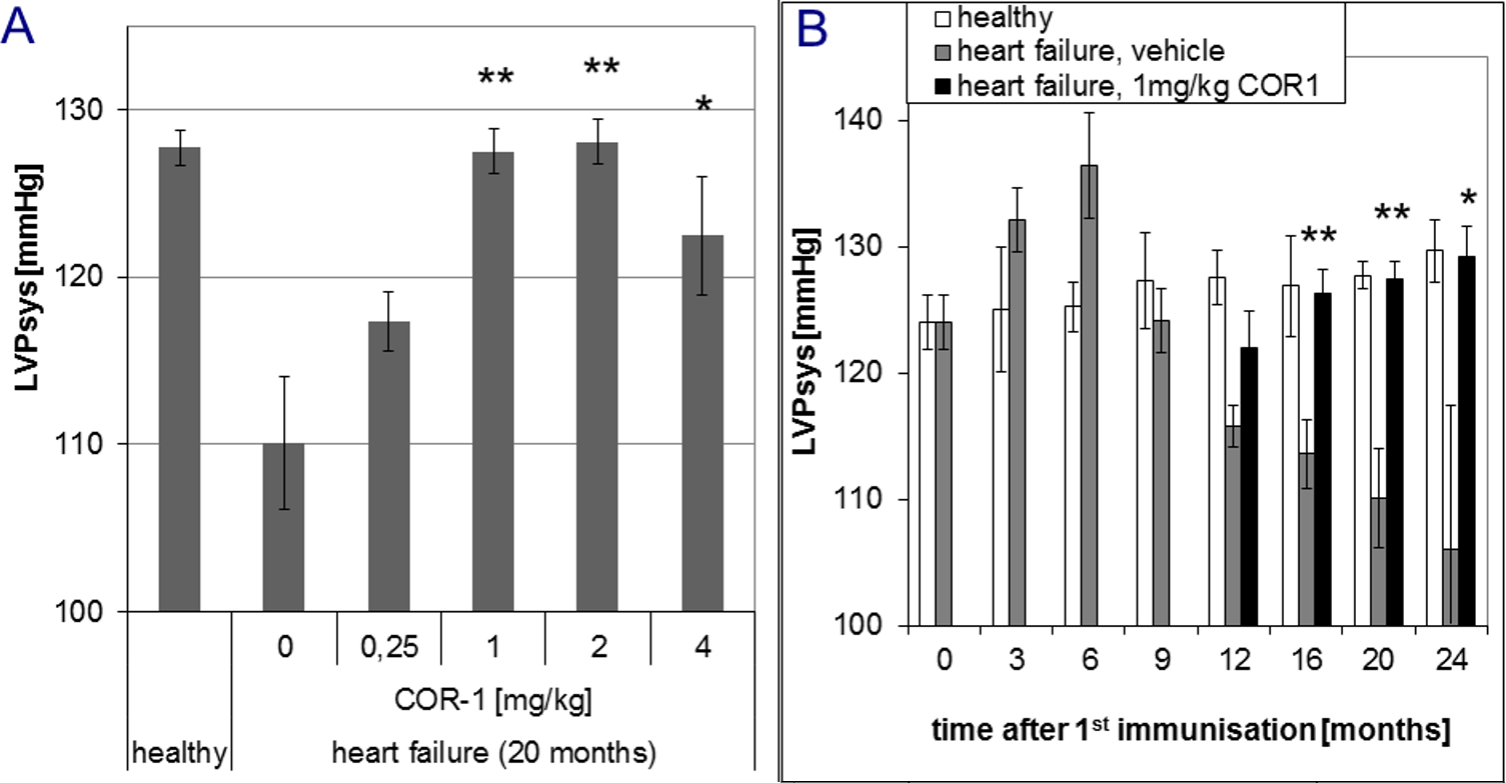
Effects of COR-1 on the left ventricular systolic pressure (LVPsys) of rats with HF induced by immunisation with GST-ß_1_EC2 fusion proteins. **A:** LVPsys of immunized rats after ten treatments(20 months after the first immunization), as determined by cardiac catheterization. Means of each group are shown with SEM for ß_1_EC2HF rats treated with vehicle (n = 5), 0.25 (n = 4), 1 (n = 10), 2 (n = 5), and 4 mg/kg BW COR-1 (n = 9), respectively. Mean LVPsys of healthy control animals are shown for comparison. *indicate statistical significance (p< 0.05) and **strong statistical significance (p<0.005), respectively, compared to the HF vehicle control group.The resulting ANOVA analyses showed overall significance, and post-hoc specific inter-group p values were 0.04 for HF + vehicle vs. healthy, and 0.002 for HF + 1 mg/kg COR-1 vs. HF + vehicle, 0.006 for HF + 2 mg/kg COR-1 vs. HF + vehicle, 0.0047 for HF + 4 mg/kg COR-1 vs. HF + vehicle. All other post-hoc analyses yielded non significant results. **B:** Time course of LVPsys, assessed every three months in healthy rats (white; n = 3 to 6) and ß_1_EC2HF rats treated 10 months after the first immunization-boostwith either vehicle (grey; n = 3 to 5) or with 1 mg/kg COR-1 (black;n = 4–10).Mean LVPsys with SEM are shown for all groups. *(p<0.05) and **(p<0.005) indicate statistical significance compared to the HF vehicle group. Analysis of variance (ANOVA) revealed no differences at baseline, but significant worsening of LVPsys in the immunized HF rats compared to healthy control rats, starting 12 months after the first immunisation. HF rats treated with 1 mg/kg COR-1 differed significantly from the HF vehicle group, but did not significantly differ from healthy control rats at 16, 20, and 24 months.

**Fig 7:**
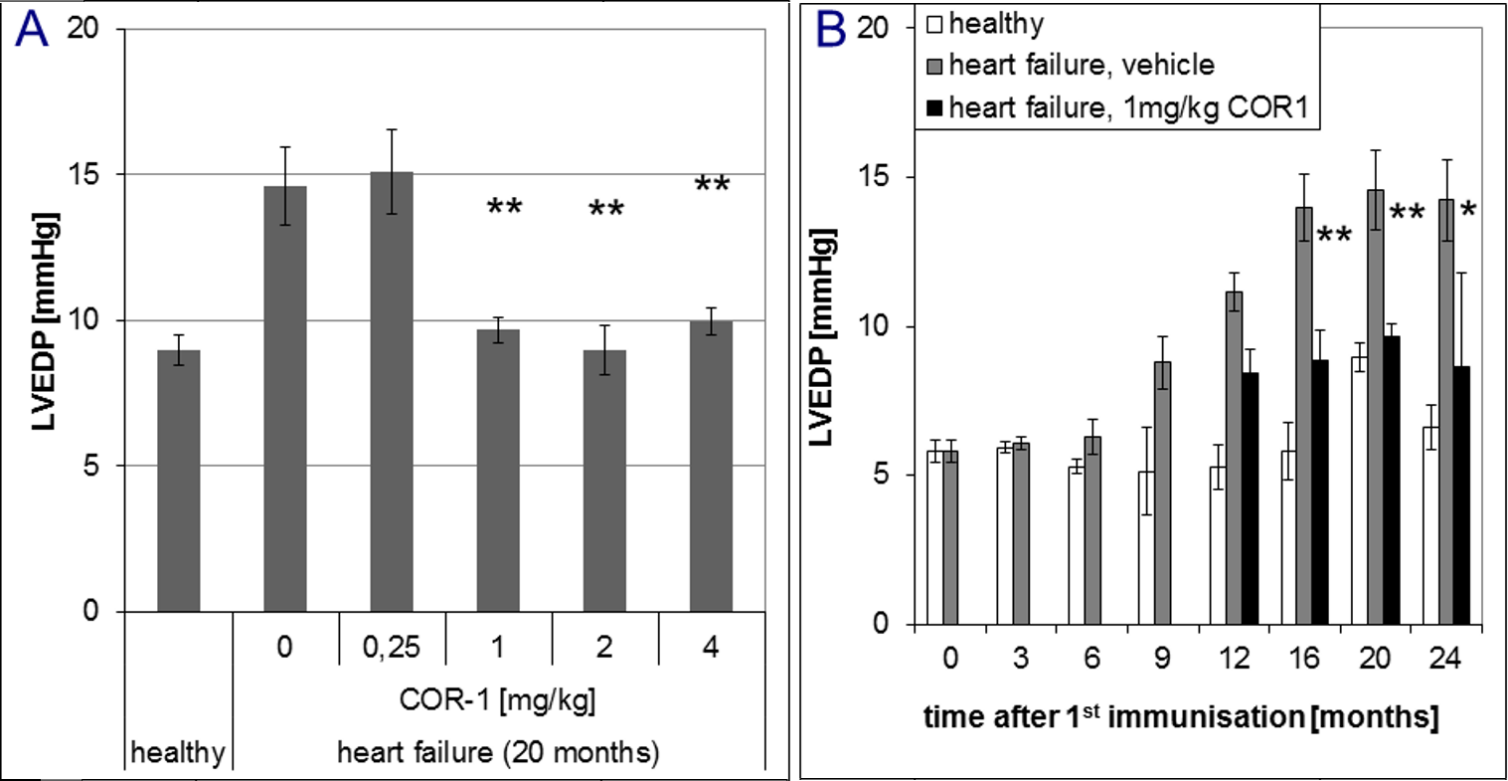
Effect of COR-1 on the left ventricular end-diastolic pressure (LVEDP) of rats with HF induced by immunisation with GST-ß_1_EC2 fusion proteins. **A:** Effects of COR-1 on the LVEDP after ten monthly treatments (20 months after the first immunization), as assessed by cardiac catheterization. Mean LVEDP (triplicate determinations in each animal) are shown with SEM for ß_1_EC2HF rats treated with vehicle (n = 5), 0.25 mg/kg BW COR-1 (n = 4), 1 mg/kg BW COR-1 (n = 10), 2 mg/kg BW COR-1 (n = 5), and 4 mg/kg BW COR-1 (n = 9), and of healthy control rats. ** indicates strong statistical significance (p<0.005) compared to the HF vehicle group. The resulting ANOVA analyses showed overall significance, and post-hoc specific inter-group p values were 0.000 for HF + vehicle vs. healthy, 0.000 for HF + 0.25 mg/kg COR-1 vs. healthy, and 0.001 for HF + 1 mg/kg COR-1 vs. HF + vehicle, 0.001 for HF + 2 mg/kg COR-1 vs. HF + vehicle, 0.002 for HF + 4 mg/kg COR-1 vs. HF + vehicle. Also, 0.001 for HF + 1 mg/kg COR-1 vs. HF + 0.25 mg/kg COR-1, 0.01 for HF + 2 mg/kg COR-1 vs. HF + 0.25 mg/kg COR-1, 0.02 for HF + 4 mg/kg COR-1 vs. HF + 0.25 mg/kg COR-1. All other post-hoc analyses yielded non significant results. **B:** Time course of LVEDP of healthy rats (white diamonds, n = 3 to 6) and of ß_1_EC2HF rats receiving vehicle (grey squares, n = 3 to 5), at intervals of three and four months. From the 12^th^ month after the first immunization on, 1 mg/kg COR-1 treated HF rats were included into the analysis (black triangles, n = 4 to 10). Mean LVEDPs with SEM are shown (n = number of respective rats).*(p<0.05) and **(p<0.005) indicate statistical significance compared to the HF vehicle group. Analysis of variance (ANOVA) showed no differences at baseline, but significant worsening of LVEDP in immunized HF rats compared to healthy control rats, starting 12 months after the first immunisation. HF rats treated with 1 mg/kg COR-1 differed significantly from the HF vehicle group, but did not significantly differ from healthy control rats at 16, 20, and 24 months.

**Fig 8:**
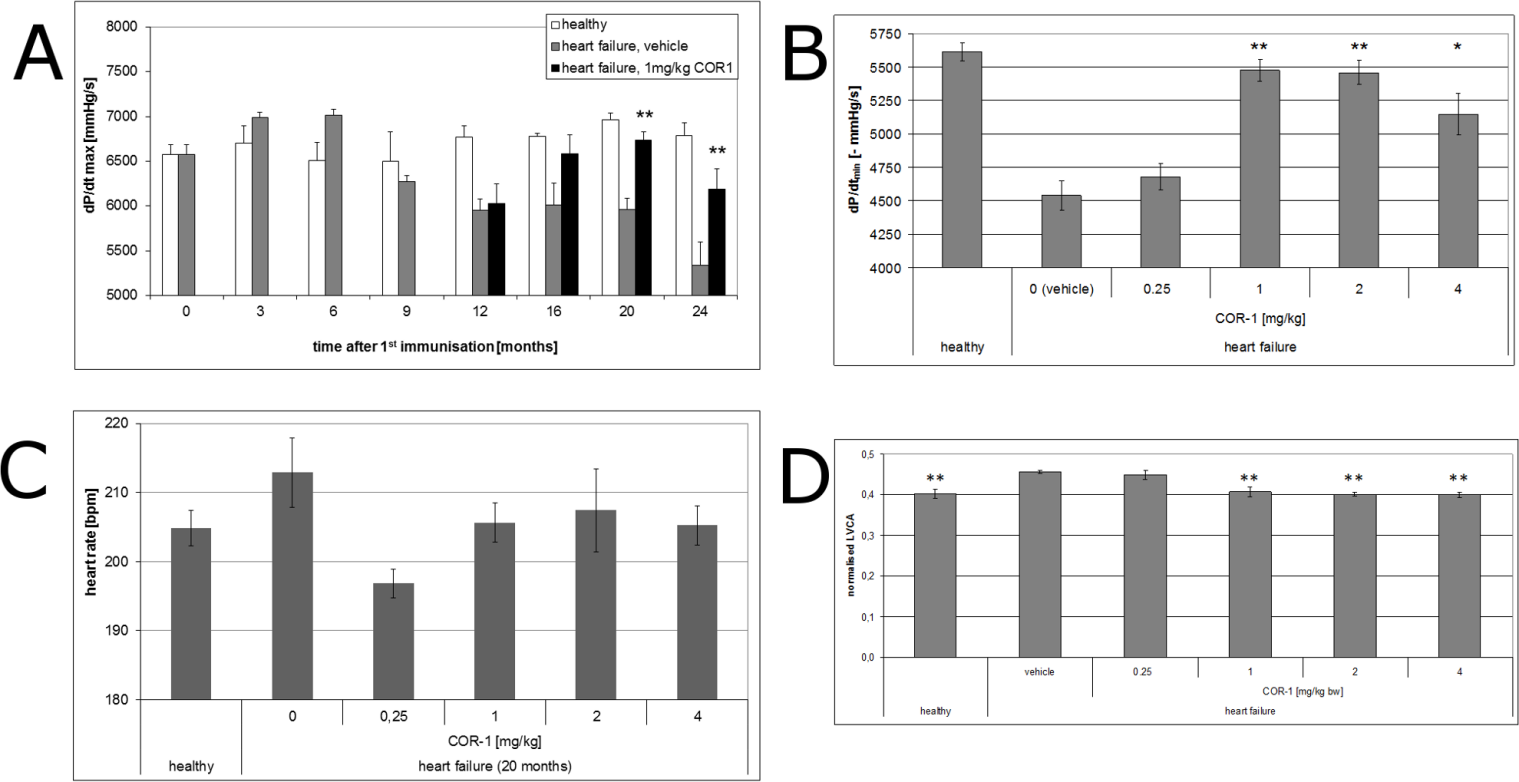
Contractility (dP/dt_max_), relaxation (dP/dt_min_), heart rates and left ventricular cavity areas (LVCA) of healthy rats and HF rats treated with either vehicle or COR-1. **A: Time course of contractility (dP/dt**_**max**_**)** The myocardial contractility of healthy rats (white diamonds, n = 3 to 6) and of ß_1_EC2HF rats receiving vehicle (grey squares, n = 3 to 5), respectively, was measured at intervals of three and four months by cardiac catheterization. From the 12th month after the first immunization on, 1 mg/kg COR-1 treated HF rats were included in the analysis (black triangles, n = 4 to 10). Mean dP/dt_max_ with SEM are shown are shown for all rats analysed at a same time point (n = number of respective rats). ** indicates strong statistical significance (p<0.005) compared to the HF vehicle group. Analysis of variance (ANOVA) revealed no differences at baseline, but significant worsening of dp/dt max in immunized HF-rats compared to healthy control ratsat the 12^th^, 20^th^ and 24^th^ month after the first immunisation. HF-rats treated with 1 mg/kg COR-1 differed significantly from the HF vehicle group, but did not significantly differ from healthy control rats at 20 and 24 months. **B: Relaxation (dP/dt**_**min**_**) of healthy rats and HF rats treated with either vehicle or COR-1** Means ± SEM of the myocardial relaxation of HF rats are shown after ten treatments (20 months after the first immunization), including the groups treated with vehicle (n = 5), or 0.25 mg/kg COR-1 (n = 4), 1 mg/kg COR-1 (n = 10), 2 mg/kg COR-1 (n = 5), or 4 mg/kg BW COR-1 (n = 9), respectively. For comparison, mean heart rates are also shown for healthy control rats (n = 6).* indicates statistical significance (p<0.05), and ** indicates strong statistical significance (p<0.005), compared to the HF vehicle group. **C: Effect of COR-1 on the heart rate of rats with HF induced by immunisation with GST-**ß_1_EC2 **fusion proteins** The effect of COR-1 on the heart rate of ß_1_EC2HF ratswas evaluated after ten monthly treatments (20 months after the first immunization). Means ± SEM of heart rates of HF rats are shown, treated with vehicle (n = 5), or 0.25 mg/kg COR-1 (n = 4), 1 mg/kg COR-1 (n = 10), 2 mg/kg COR-1 (n = 5), or 4 mg/kg BW COR-1 (n = 9), respectively. For comparison, mean heart rates are also shown for healthy control rats (n = 6). No significant differences were observed.The mean heart rate of the rats did not differ significantly between the groups (p = 0,173 by ANOVA). **D: Left ventricular cavity areas (LVCA) of healthy rats and HF rats treated with either vehicle or COR-1** Means ± SEM of the normalized LVCA (ratio of LVCA divided by the respective left ventricular area) of HF rats after ten treatments (20 months after the first immunization), including the groups treated with vehicle (n = 5), or 0.25 mg/kg COR-1 (n = 4), 1 mg/kg COR-1 (n = 10), 2 mg/kg COR-1 (n = 5), or 4 mg/kg BW COR-1 (n = 9), respectively. For comparison, mean normalized LVCAs are also shown for healthy control rats (n = 6).** indicates strong statistical significance (p<0.005 by ANOVA), compared to the HF vehicle group.

In overt disease, six i.v. administrations of 1, 2 or 4 mg/kg body weight COR-1 every month almost fully reversed the dilative cardiomyopathic phenotype, whereas 0.25 mg/kg had no effect (Fig 4A). Further treatments resulted in sustained therapeutic effects of 1, 2 or 4 mg/kg COR-1. Fig 4B shows that the beneficial effects of 1 mg/kg COR-1 increased over time and achieved a maximum 6 to 8 months after start of therapy. With 1–4 mg/kg COR-1, echocardiographic LV fractional shortening (FS, Fig 5A) was also markedly improved; Fig 5B shows the time course. Invasive cardiac parameters obtained by cardiac catheterisation at defined time points (e.g. 12, 16, 20, and 24 months) are shown in Figs 6–8, and corroborate the results obtained by echocardiography: 1–4 mg/kg COR-1 significantly improved LV systolic pressures (LVPsys, 20 months and sequential invasive measurements; Fig 6A and 6B, respectively) and lowered LV end-diastolic filling pressures which are known to be increased in heart failure (LVEDP, 20 months and sequential invasive measurements; Fig 7A and 7B, respectively). In parallel, left ventricular contractility (dp/dt max; Fig 8A) as well as ventricular relaxation improved (dp/dt min, see Fig 8B). In contrast to treatment with cardioprotective ß-blockers in patients or in the present Lewis rat model[20], basal heart rate was not altered with COR-1 (Fig 8C). Also, the heart weights of cardiomyopathic rats and the anatomic cardiac dimensions (e.g., normalized LV-cavity area,) were almost reversed to control values (see Fig 8D).

### Immunomodulating effects of COR-1

**Fig 9:**
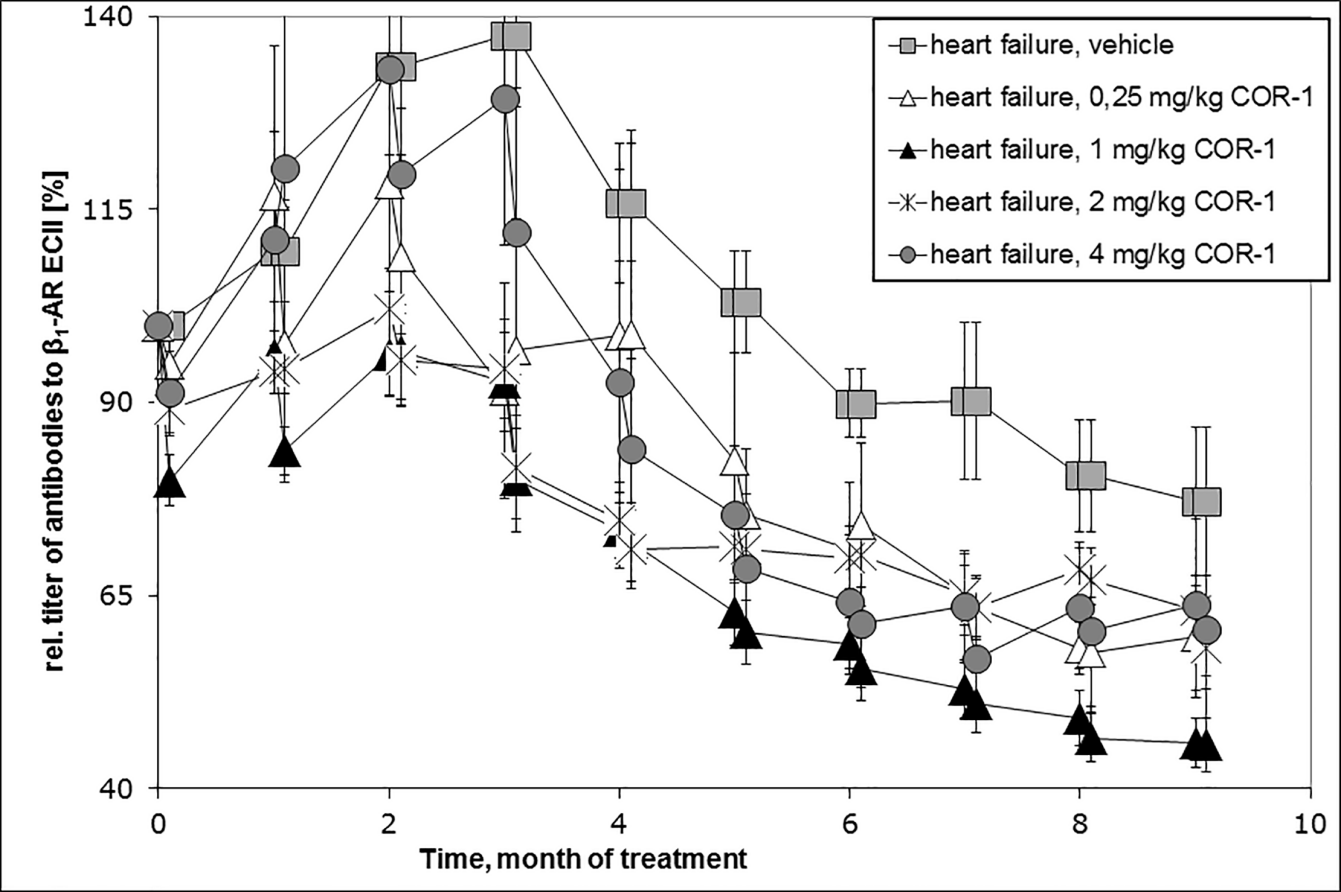
Anti-ß_1_EC2 antibody titersinduced by immunisation with GST-ß_1_EC2 fusion proteins. The time courses of the mean relative antibody titers after start of treatment as determined by ß_1_EC2 ELISA are shown for the HF vehicle group (grey squares, n = 9) and the COR-1 treatment groups receiving 0.25mg/kg (white triangles, n = 4), 1 mg/kg (black squares, n = 20), 2 mg/kg (black cross, n = 5), and 4 mg/kg BW COR-1 (grey circle, n = 9) as bolus injections, respectively. The respective data points are grouped to better visualise the titer courses, however, this does not mean that they reflect the real course between the data points. The black arrows mark time points of boosts (applications of GST-ß_1_EC2), the white arrows mark time points of treatment (COR-1 or vehicle). N = number of plasma samples from individual rats.Analysis of variance revealed no significant titer differences between the groups at start of therapy (12 months after the first immunization), but significant titer decreases in the groups treated with 1 and 4 mg/kg COR-1 compared to the HF vehicle group after 16 to 20 months of therapy. The titer decrease did not differ significantly between HF rats treated by vehicle compared to animals treated with 0.25 mg/kg and 2 mg/kg COR-1.

The scavenger effect of COR-1 on anti-ß_1_EC2 abs was less pronounced than for previously studied cyclic peptides[20]. As shown in Fig 9, after three i.v. administrations anti-ß_1_EC2–titers tended to decrease in response to therapy with COR-1 compared to the titers at initation of therapy, but this trend did not reach statistical significance at any dosing. A similar trend was observed in the control group.

**Fig 10:**
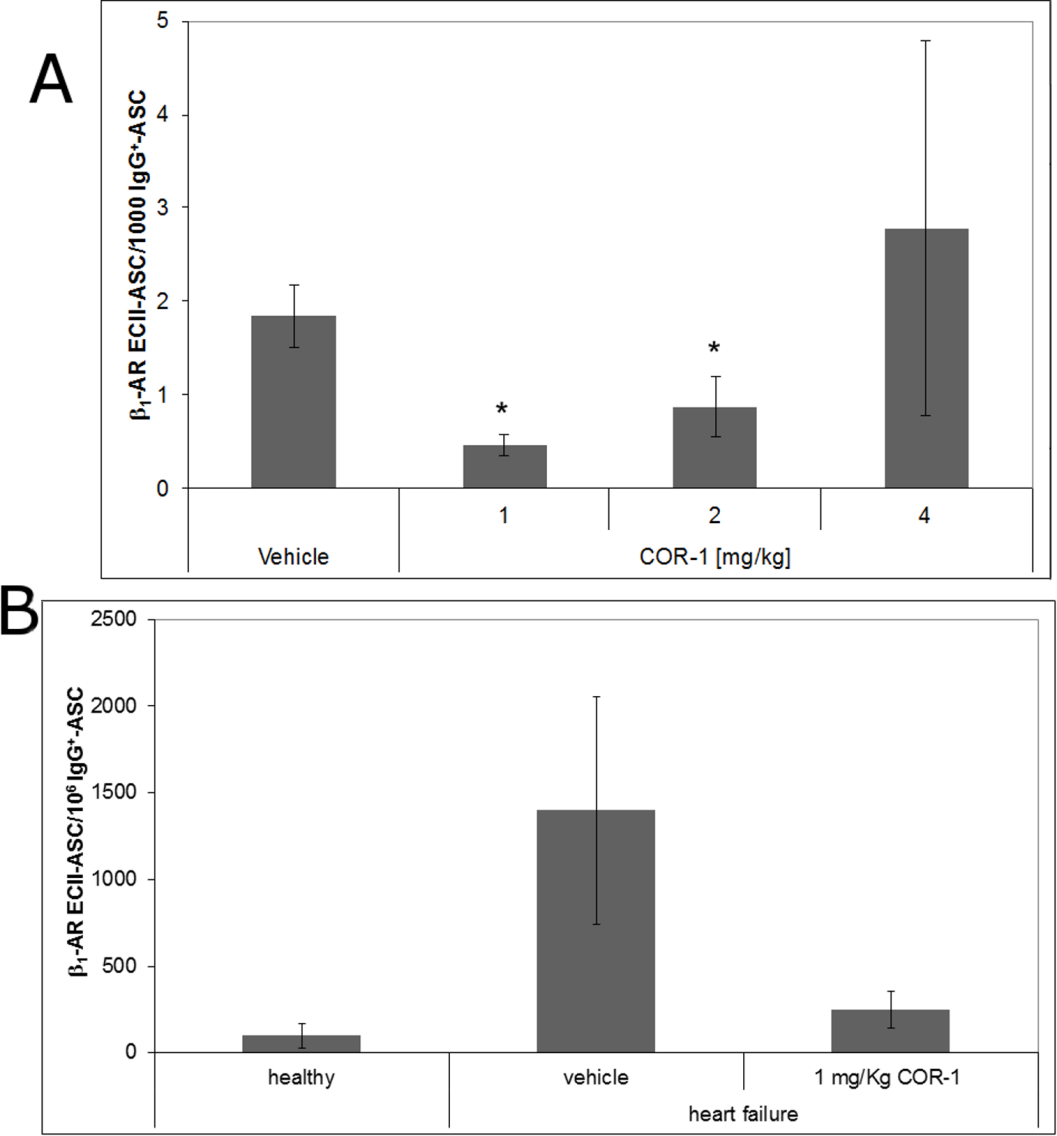
**A: Effect of COR-1 on** ß_1_EC2-**specific IgG positive B cells of the spleen of HF rats determined by ELISpot analysis (20 month).** For ELISpot analysis, spleen cells were prepared from HF rats that had received 10 monthly treat-ments with either vehicle (n = 3), or COR-1 at doses of 1 mg/kg (n = 5), 2 mg/kg (n = 3), and 4 mg/kg (n = 3), respectively. Spleen cells were incubated on plates either coated with IgG, GST, or ß_1_EC2 fusion proteins, respectively. The relative numbers of ß_1_EC2-specific IgG positive B cells were determined by subtracting the number of GST specific memory B cells from the number of ß_1_EC2-specific IgG positive B-cells and subsequent normalization to IgG^+^ B cells. Means± SEM are shown. N = number of rats analyzed per group. The values for each rat are means of two independent experiments. *p < 0.05 vs. vehicle group. **B: Effect of COR-1 on the** ß_1_EC2-**specific IgG positive B cells in the spleens of HF rats immunized against GST-**ß_1_EC2 **fusion proteins** Spleen cells prepared from healthy rats (n = 3) and from HF rats which received 10 monthly treatments with either vehicle (n = 4) or 1 mg/kg COR-1 (n = 4) were blocked with GST, stained with anti-IgG (Fc) and DyL649-labeled GST-ß_1_EC2 fusion proteins, and then analyzed by FACS. The panel depicts the ratio of specific anti-ß_1_EC2 IgG-positive cells per 1.000.000 IgG-positive B cells.Due to the large variation in the vehicle group, comparisons with the treatment groups failed to reach statistical significance.

Differential analysis of the T cell compartment of treated animals indicated that neither regulatory CD4^+^ T-cells nor other mechanisms of (suppressor-)CD8^+^ T-cells were directly involved in the effects of ß_1_EC2 25-meric cyclic peptides[20]; in contrast, antigen-specific splenic B lymphocytes were markedly reduced in treated animals[20]. ELISpot analysis of splenic B lymphocytes prepared from rats treated with 1 and 2 mg/kg COR-1 also revealed significant reduction in specific anti-ß_1_EC2–secreting B-cells (ASC) compared to vehicle control (Fig 10A). Further analysis of the B-cell compartment indicated that long-lasting anti-ß_1_EC2–specific plasma cells in the bone marrow do apparently not represent the target of COR-1 (data not shown).

Another study carried out in rats of the same series, which had either received vehicle or 1 mg/kg body weight (BW) of COR-1, used direct FACS analysis of splenic B cells instead of ELISpot to trace antigen-specific plasma cells. FACS-data revealed a reduction of these B cells by more than 80% after 10 monthly treatments in anti-ß_1_EC2–positive rats (Fig 10B). However, a FACS-analysis of the other COR-1 dose groups was not possible due to smaller animal numbers/group and thus scarcity of isolated splenic immune cells.

### Effect of COR-1 on cardiac ß_1_-AR mRNA levels

**Fig 11:**
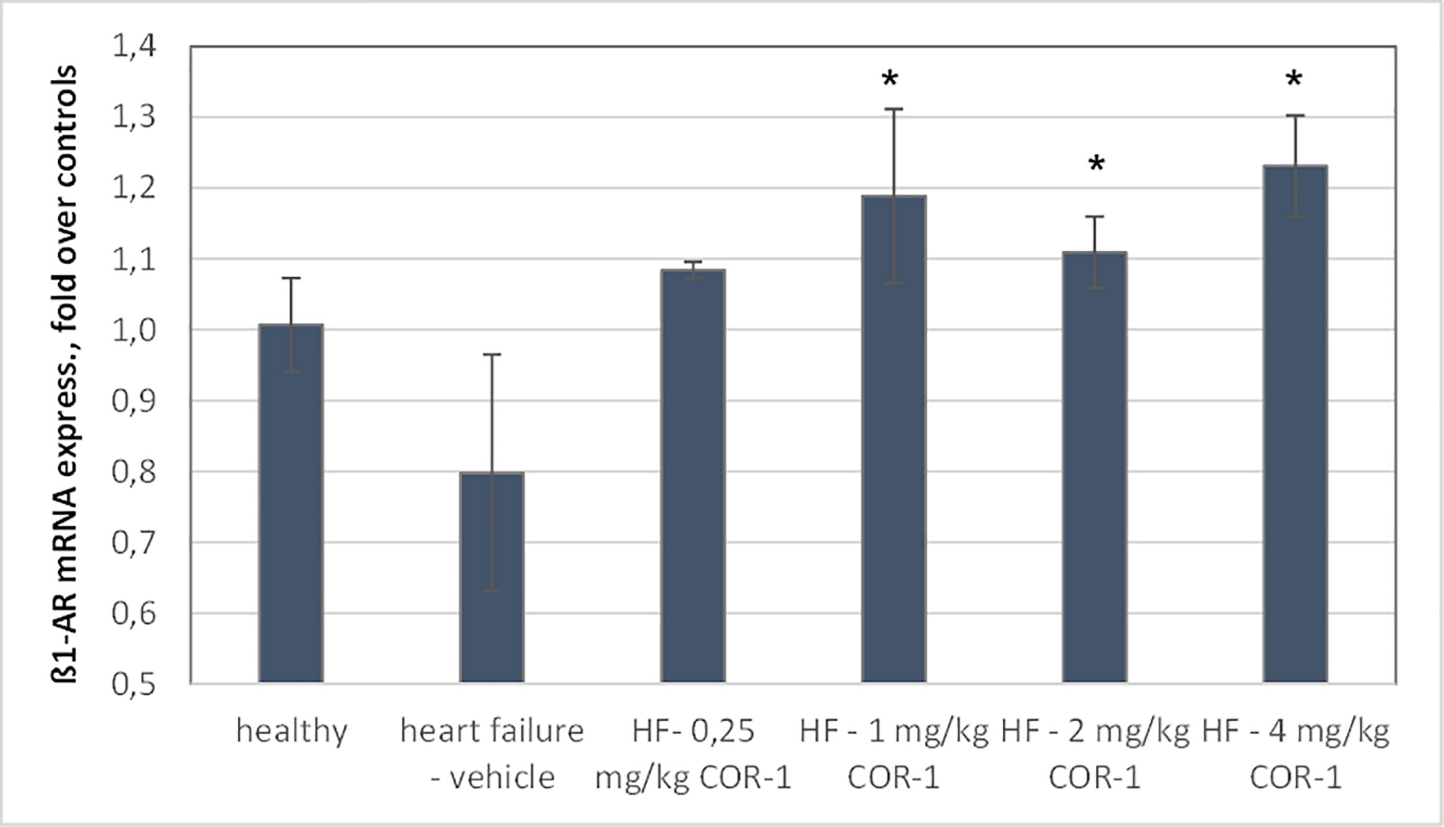
Cardiac mRNA levels of ß_1_-adrenergic receptors (ß_1_-AR) of healthy rats and HF rats treated with either vehicle or COR-1. Means ± SEM of the normalized ß_1_-AR mRNA (ratio of ß_1_-AR R mRNA divided by the respective GAPDH mRNA level) of HF rats after ten treatments (20 months after the first immunization), including the groups which had been treated with vehicle (n = 5), or 0.25 mg/kg COR-1 (n = 4), 1 mg/kg COR-1 (n = 10), 2 mg/kg COR-1 (n = 5), or 4 mg/kg BW COR-1 (n = 9), respectively. For comparison, mean normalized ß_1_-AR mRNA levels are also shown for healthy control rats (n = 6). * indicates statistical significance (p<0.05 by ANOVA), compared to the HF vehicle group.

Myocardial mRNA expression was investigated in rats after the end of the study. Fig 11 shows that treatment with 1–4 mg/kg COR-1 resulted in significantly increased ß_1_-AR mRNA levels, compared to the non-treated HF group.

### Effect of COR-1 on plasma cytokine levels

**Fig 12:**
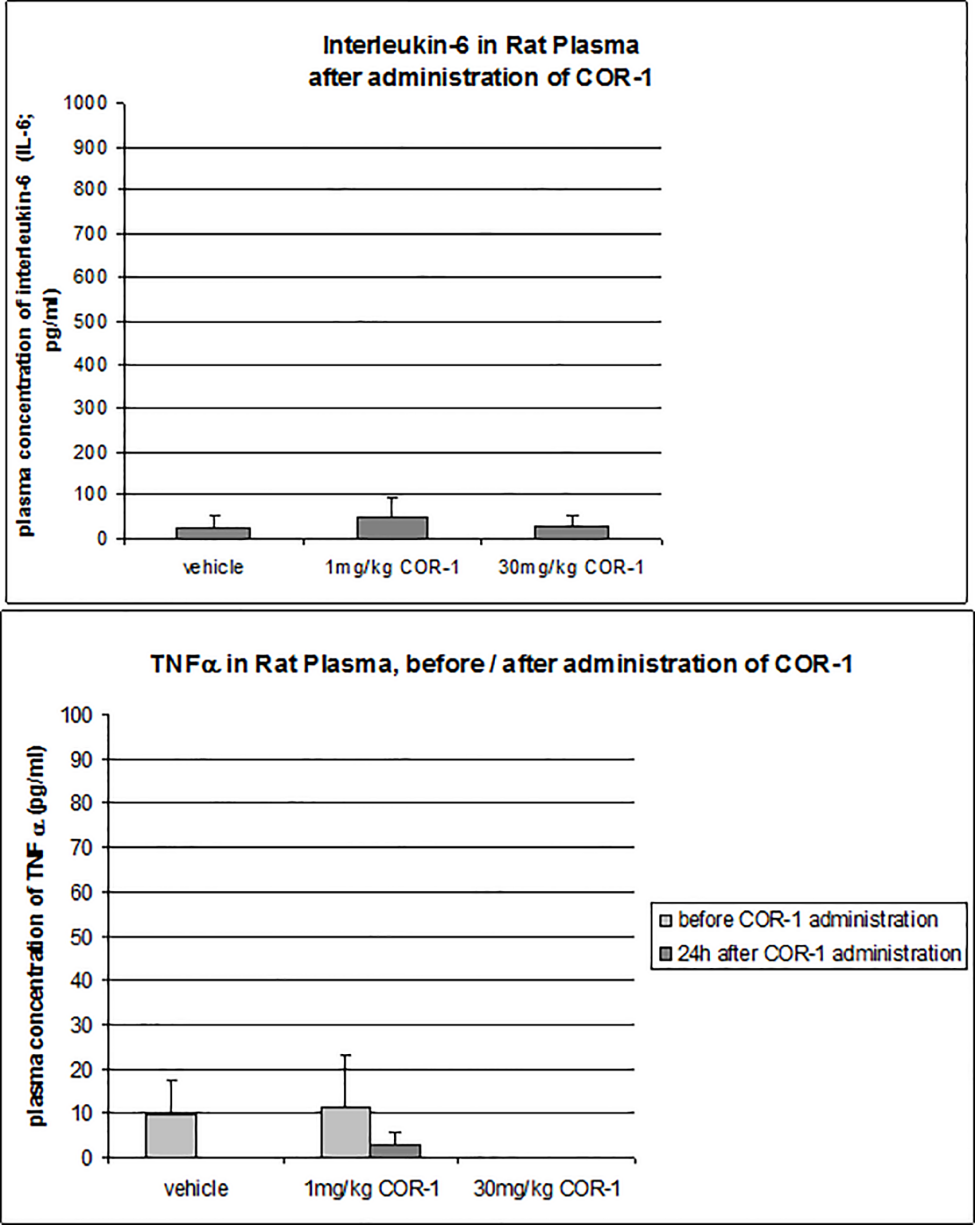
Effect of COR-1 on the plasma levels of interleukin (IL)-6 and tumor necrosis factor (TNF)-alpha. Spleen cells prepared from HF rats which received 10 monthly treatments with either vehicle (n = 8) or 1 mg/kg or 30 mg/kg COR-1 (n = 8 each) were assessed for plasma levels of IL-6 after treatment (A) and TNF-alpha before and after treatment (B).There were no significant differences between groups.

In the frame of the present study we also investigated the effects of COR-1 on cytokines which are known for being activated on the short term. After 7-fold administration of GST-ß_1_AR fusion protein, 1 mg/kg BW or 30 mg/kg BW COR-1 or vehicle was given to anti-ß_1_EC2 positive male Wistar rats by i.v. bolus injection (n = 8 rats per group). 24 hours thereafter, blood samples were taken from all animals, and interleukin-6 (IL-6) concentration was determined by ELISA. No differences in IL-6 levels were observed between the groups: Please see results in Fig 12A.

Similarly, no differences were detected in plasma tumor necrosis factor (TNF-alpha) levels between the groups (measurements in n = 8 independent animals in each group). Most values were near or below the limit of detection, i.e. at very low values. Please see results in Fig 12B.

### Investigation of immunologically naïve animals

The effects of COR-1 were also assessed in naïve, non-immunized rats to exclude antigenecity or general immune responses to COR-1. Male Lewis HanHsd rats were treated with 0.25 to 5 mg/kg COR-1 by i.v. bolus injection every 4 weeks, for a total of six months. Six rats were included in each group and assessed independently. Blood samples were taken prior to as well as 24h andtwo weeks after COR-1 injections, respectively, and analyzed for anti-COR-1 or anti-ß_1_EC2titers by ELISA.

The sensitivity for the detection of anti-ß_1_AR abs was assessed by employing known concentrations of the monoclonal anti-ß_1_EC2antibody 23-7-6[22] and was determined to be 660 pmol/L. Injection of COR-1 did neither result in the generation of anti-ß_1_EC2abs nor of anti-COR-1 abs in any of the treated animals over an observation period of six months. In addition, the presence of specific anti-COR-1 IgM was analysed by using anti-rat IgM-specific antibodies. In COR-1 treated animals no such antibodies could be detected at any time.

### Toxicological investigations and further safety studies in dogs and rats

The effects of COR-1 at toxicological dose escalations were assessed in rats and dogs. Table 1 shows an overview on the most relevant studies and their results. No toxicity was observed at up to 100-fold dose escalation in rats or dogs, over a period of six months.

**Table 1:** Most relevant toxicology studies with COR-1.

study#	species/strain n/group	duration route	dose (mg/kg)	C_max_ (nmol/L)	AUC_0-t_ (nmol.min/L)	notewor. observ.	NOAEL safety margin to starting dose
GLP safety study 2: Harlan 10-4-0155-07	rats (Wistar) 6 males 6 females	28 days repeated	25	66 199	184 211	none	NOAEL > 100 mg/kg
		i.v.	50	105 695	431 412	none	HED > 16 mg/kg BW
			100	221 000	1 214 000	none	safety marginto starting dose (0.14 mg/kg) = 114
GLP safety study 3: Aurigon 433.123. 1635	dogs (Beagles) 3 males 4 females	14 daysrepeated;	10	29 653	134 881	none	NOAEL > 100 mg/kg
		14 daysrecovery	20	63 428	223 048	none	
		i.v. bolus	30	87 129	437 299	none	HED > 55 mg/kg BW
GLP safety study 4: Rds Hameln 60072410	dogs (Beagles) 4 males 4 females	single i.v. infusion	100	n.a.	2 062 000	none	safety margin to starting dose (0.14mg/kg) = 392
		6 months repeated;	7,5	15 171	70 830	none	NOAEL > 30 mg/kg
		2 monthsrecovery	15	41 428	205 933	none	
		i.v. bolus	30	82 830	514 470	none	

Conversion factor according to FDA Guidance (to convert animal dose to Human Equivalence Dose; HED): dog: 1.8, rat: 6.2

In safety study 1, no clinical abnormalities were observed, and gross investigation of the animals after necropsy did not reveal any pathologies. Furthermore, thorough histopathological examination of tissue sections of the mandibular lymphatic nodes, trachea and lung with bronchi, heart, thoracic aorta, spleen, liver, adrenal glands and kidneys did not reveal any pathological effects of a treatment with COR-1. Analysis of haemodynamic parameters obtained by cardiac catheterisation revealed a favourable effect of COR-1 treatment on heart failure development in anti-ß_1_EC2–positive rats. The mean myocardial contractility improved significantly when compared to the vehicle-treated cardiomyopathic control rats. Furthermore, treatment with COR-1 yielded a trend towards improved myocardial relaxation together with normalization of LVPsys and LVEDP.

In safety study 2, no adverse events and also no gross macroscopic or microscopic abnormalities of any organs were observed in the animals. Analysis of blood samples from all animals revealed no relevant alterations between COR-1 or vehicle groups.

In safety study 3, neither ECG & blood pressure recordings, clinical examinations, macroscopic and microscopic investigations, nor analysis of haematology or standard clinical chemistry revealed any abnormal findings.

In safety study 4, one animal of the medium dose group, receiving 15 mg/kg COR-1, experienced an episode of reduced, almost absent food consumption for three days, starting 21 days after the fifth administration of COR-1 (10.080 half-lives after the last drug administration), which was accompanied by an increase in white blood cell count up to 24.800/μm^3^ (normal values < 14.000/μm^3^) but no deviations in any other routine laboratory parameters. After 3 days, the animal fully recovered; white blood cell count normalized and remained sable until study-end. As a consequence, this event was attributed to a non-specific intercurrent infection. No other adverse events were reported for any of the other study-animals. Body weights and food consumption developed equally and normally in all groups. No pathological clinical signs or symptoms were observed on daily examinations, or during ophthalmoscopy or measurements of blood pressure, pulse rate and ECG. Also, routine laboratory (haematology and standard clinical chemistry) revealed no abnormalities. No gross pathologies of any organs were observed upon macroscopic investigation; 42 organs from each animal were also carefully analysed by histology, yielding no pathological microscopic findings.

### Safety pharmacology

In summary, there was no evidence for any cardiovascular, respiratory, renal or central (CNS) side effects of COR-1 at doses of up to 30 mg/kg body weight in rats, guinea pig, or mice, and up to 100 mg/kg body weight in dogs (see Table 2).

**Table 2:** Safety pharmacology studies.

study number	evaluated systems	species strain	route of admin.	doses	number gender	GLP compliance	noteworthy findings
Aurigon 433.520.1669 433.123.1635	cardio- vascular	dogs beagles	IV	15–100 mg/kg	3M, 3F	yes	none
				10–30 mg/kg	11M, 14F	yes	none
Harlan 10-4-0105-08	cardio- vascular	rats Wistar	IV	30 mg/kg	5M, 5F	yes	no biologically relevant findings
inhouse safety study	cardio- vascular	rats Wistar	IV	30 mg/kg	10M	no	none
Harlan 10-4-0104-08	respiratory	guinea pigs Dunkin	IV	30 mg/kg	3M,3F	yes	none
Harlan 10-4-0107-08	CNS	mice NMRI	IV	15 mg/kg	5M,5F	yes	none
Harlan 10-4-0106-08	renal	rats Wistar	IV	15 mg/kg	3M,3F	yes	none

A human Ether-a-go-go related gene (hERG) Channel was performed by patch clamping of CHO cells stably expressing hERG. No effect on hERG channel activity was observed with 10 μg/ml, 100 μg/ml or 1 mg/ml COR-1 (corresponding to the serum levels observed after i.v. bolus administration of 1 mg/kg COR-1 in both rats and dogs, or a 10- and 100-fold dose escalation), whereas the positive control experiment using E4031 (100 nM) yielded the expected ion channel currents.

The effect of COR-1in doses of 15, 30, 100 mg/kg or vehicle on cardiovascular function was investigated in conscious Beagle dogs (three male and three female). COR-1 was applied in ascending doses, followed by a wash-out period of 72 hours. Telemetered diastolic, systolic and mean blood pressures were recorded up to one hour post dosing, revealing no significant blood pressure changes. No abnormal clinical findings and no life-threatening effects were observed. A full ECG documentation of Beagle dogs during i.v. infusion of COR-1 yielded no abnormalities. Therefore, the NOAEL level regarding cardiovascular effects is above 100 mg/kg BW in dogs. Moreover, the effects of COR-1 (30 mg/kg) or vehicle on the cardiovascular function of conscious Wistar HsdHan:WIST rats (five male and five female) was investigated (systolic, diastolic, and mean blood pressure, heart rate, ECG). No biologically relevant adverse effects occurred.

The effect of COR-1 (30 mg/kg) or vehicle on the respiratory function was investigated of three male and three female conscious guinea pigs. Assessment of respiratory function including respiration frequency, tidal volume and minute volume revealed no adverse effects attributable to COR-1.

Finally, the effects of 15 mg/kg COR-1 on the central and autonomic nervous system of NMRI HsdWin mice were assessed by clinical observation according to the IRWIN screen procedure, and by analysing psycho-motor behaviour. In five male and five female mice,no adverse effects could be observed; in addition, no pathological findings were recorded at any time during the IRWIN screen.

## Discussion

Autoantibodies directed against self-antigens occur in many autoimmune diseases, and often may even cause the disease[23]. Particularly, in Graves‘ disease[24], and—more recently—also in anti-ß_1_AR-induced autoimmune cardiomyopathy[6,20], functionally active autoantibodies directed against membrane receptors have been recognized as main pathogenetic factors.

ß adrenergic receptors and associated G protein coupled receptor kinases (GRKs) play an important role in heart failure[25–27]. In cardiomyopathic patients, the presence of conformational activating anti-ß_1_EC2–abs has been associated with a more severely depressed cardiac function[^11^], the occurrence of more severe ventricular arrhythmias[12], a higher incidence of sudden cardiac death[^12^], and with an increased cardiovascular mortality risk[13]. Thus, the available clinical data underscore the pathophysiologic and clinical importance of stimulating anti-ß_1_EC2–abs in heart failure, and the need for novel specific antibody-directed therapeutic strategies[6]. Current treatment approaches in autoantibody-mediated diseases comprise administration of anti-CD20antibodies, immunoadsorption or glucocorticosteroids and/or cyclo-phosphamide. These strategies are aggressive, time- and cost-consuming, and bear a high risk for severe side effects together with an uncertain outcome.

We and others have previously shown that immunization against the second EC-loop of the human ß_1_-AR gives rise to catecholamine-like acting anti-ß_1_EC2–abs in various animal models[17,20]. In the heart, such antibodies are supposed to allosterically activate the adrenergic signaling cascade even in the absence of catecholamines[6,20], and in the long run cause myocardial tissue injury, myocyte apoptosis, fibrosis, and finally congestive heart failure[6,7]. The most likely explanation is that anti-ß_1_EC2–abs mildly but chronically activate cardiac membrane ß_1_AR and, thus, either initiate or potentiate the vicious circle of sympathetic overdrive and heart failure progession[6,7].Anti-ß_1_AR antibodies do not exert strong positive chronotropic effects in adult Lewis rats, as shown before in the same model[20], probably because ß_1_-AR activation impacts predominantly on contractility, whereas basal heart rate is regulated by ß_2_-adrenergic receptors in adult rats[28,29].

In this human-analogous Lewis rat model, monthly injection of COR-1 resulted in almost complete reversal of heart failure. Monthly injections of COR-1 were well tolerated by both immunized anti-ß_1_EC2–positive rats and antibody-naïve control animals, and during one year of regular treatment elicited no serious side effects. Cardioprotection achieved with monthly COR-1 injections was superior to daily applications of bisoprolol [20], which only delayed progression of anti-ß_1_EC2–induced heart failure. Unlike bisoprolol, COR-1 neither affected heart rate nor blood pressure. The observed trend towards reduced heart rates with 0.25 mg/kg COR-1 was not significant and can be regarded as circumstantial variation without further meaning.Previous studies in the same rat model showed an upregulation of cardiac ß-adrenergic receptor densities and mRNA levels in response to IV therapy with predecessor cyclic peptides. Also treatment with COR-1 resulted in significant upregulation of cardiac ß_1_-AR mRNA levels. Since COR-1 does not interact with cardiac ß_1_-adrenoceptors directly, this finding should reflect a general myocardial recovery which can be effectuated by such a treatment.

After 4 injections of COR-1, anti-ß_1_EC2titers remained stable in spite of continued monthly antigen boosts. The effect occurred more or less pronounced in all COR-1-treated groups, so that we can only speculate on the potential inhibitory effects of COR-1 on the anti-ß_1_EC2 specific B cells in the spleens of treated animals. From our ELISpot and FACS analysis of splenic cells we conclude, however, that COR-1 might have acted as an inhibitor of the ß_1_EC2–specific B cell receptor (BCR). In that case, COR-1 would address the cause of the disease without adverse immunologic effects: by binding to the ß_1_EC2-specific BCR as soluble monovalent antigen, COR-1 might hinder further antigen-mediated crosslinking of the BCR, impeding BCR-triggered B cell expansion or actively inducing apoptosis in ß_1_EC2-specific memory B cells[20]. Thus, in the spleens of immunized rats treated with COR-1, blockade/monomeric stimulation of the ß_1_EC2-BCR may have resulted in the observed substantial reduction in anti-ß_1_EC2 IgG antibody-producing memory B cells. It should be noted, however, that the non-ß_1_EC2–presenting cells, that is, other IgG producing B cells in the spleen or circulation involved in the adaptive humoral response were not affected in COR-1-treated immunized animals. Thus, a general immunosuppressive effect of COR-1 can almost be excluded.

Since anti-ß_1_EC2-mediated cardiostimulatory effects cannot be efficiently neutralized with ß_1_-receptor blockers alone[9,20], we initiated a clinical development program in which we showed that COR-1 is safe in human volunteers[30]. A first phase II study resulted in encouraging results for heart failure patients treated with 1 mg/kg COR-1 i.v. every four weeks over 6 months[31]with no safety concerns, encouraging further assessment of tailored cyclic peptides for the treatment of anti-ß_1_AR autoantibody-positive human heart failure in larger clinical trials. These studies also reconfirmed renal and liver safety. A phase Ib study investigated shorter dosing intervals[32]–using this unusually short dosing interval regime, two participants with predisposing risk factors (including a factor V Leiden mutation) had thromboembolic adverse events; further detailed analysis including functional tests revealed neither pro-coagulatory nor anti-coagulatory substance-related effects of COR-1 *per se*. Other experimental antibody-directed strategies consist in their removal from the circulation by specific or non-specific immunoadsorption using either matrix-coupled peptides derived from ß_1_EC2–[33]or protein A columns[34]. A recent meta-analysis showed promising results with excellent survival rates after treatment with specific matrix-coupled columns with peptides derived from ß_1_EC2[35]. However, this approach is expensive, time-consuming, and still needs to be validated in a larger randomized still ongoing prospective clinical trial[36]. Another approach relies on an inactivation of receptor-autoantibodies by aptamers[37], and is currently tested in a phase I clinical study. Whilst the aptamer-approach uses i.v. application of small DNA-fragments interacting with the respective autoantibodies (and, thus, appears similarly “easy-to-use” as has been demonstrated for COR-1), the *in vivo* effect and efficacy of aptamers in patients still needs to be explored. In the meantime, the here presented concept of tailored epitope-mimicking cyclic peptides to treat anti 7TM-receptor-directed autoimmune diseases has been recently extended to an experimental treatment of Graves' disease[38].

Conclusion: In addition to previous work[^18^] on ß_1_EC2–mimicking cyclic peptides to treat autoimmune-mediated heart disease by scavenging cardio-noxious anti-ß_1_AR autoantibodies and by modulating the activity of ß_1_EC2–specific pre-B cells, we here present the effects and thorough pre-clinical characterization of a shortened, slightly modified, and better producible variant, termed COR-1. Application of COR-1 might represent acost-saving, easy-to-use, and safe novel therapeutic approach for patients suffering from autoimmune heart failure.
